# High-Resolution SEM and EDX Characterization of Deposits Formed by CH_4_+Ar DBD Plasma Processing in a Packed Bed Reactor

**DOI:** 10.3390/nano9040589

**Published:** 2019-04-10

**Authors:** Mohammadreza Taheraslani, Han Gardeniers

**Affiliations:** 1 Mesoscale Chemical Systems, MESA+ Institute for Nanotechnology, University of Twente, P.O. Box 217, 7500 AE Enschede, The Netherlands; j.g.e.gardeniers@utwente.nl; 2 Faculty of Science and Technology, University of Twente, P.O. Box 217, 7500 AE Enschede, The Netherlands

**Keywords:** plasma catalysis-methane conversion, dielectric barrier discharge, deposits, materials characterization

## Abstract

The deposits formed during the DBD plasma conversion of CH_4_ were characterized by high-resolution scanning electron microscopy (HRSEM) and energy dispersive X-ray elemental analysis (EDX) for both cases of a non-packed reactor and a packed reactor. For the non-packed plasma reactor, a layer of deposits was formed on the dielectric surface. HRSEM images in combination with EDX and CHN elemental analysis of this layer revealed that the deposits are made of a polymer-like layer with a high content of hydrogen (60 at%), possessing an amorphous structure. For the packed reactor, γ-alumina, Pd/γ-alumina, BaTiO_3_, silica-SBA-15, MgO/Al_2_O_3_, and α-alumina were used as the packing materials inside the DBD discharges. Carbon-rich agglomerates were formed on the γ-alumina after exposure to plasma. The EDX mapping furthermore indicated the carbon-rich areas in the structure. In contrast, the formation of agglomerates was not observed for Pd-loaded γ-alumina. This was ascribed to the presence of Pd, which enhances the hydrogenation of deposit precursors, and leads to a significantly lower amount of deposits. It was further found that the structure of all other plasma-processed materials, including MgO/Al_2_O_3_, silica-SBA-15, BaTiO_3_, and α-alumina, undergoes morphological changes. These alterations appeared in the forms of the generation of new pores (voids) in the structure, as well as the moderation of the surface roughness towards a smoother surface after the plasma treatment.

## 1. Introduction

SEM/EDX studies have been extensively utilized to analyse the surface morphology of catalyst samples. The surface morphology of a catalyst can play a key role in determining the activity, selectivity, and stability of the catalyst. In recent years, low-temperature non-thermal plasma systems have been exploited for the preparation and treatment (e.g., reduction) of packing materials and metal-supported catalysts, advantageously preferred to conventional thermal approaches, which demand a quite high operating temperature for the preparation and treatment (e.g., calcination, reduction) of catalysts [1,2,3].

Plasma treatment of the catalyst can result in effective changes in the morphology and catalytic properties of the surface, depending on the amount of applied power, the exposure time, and the type of gas, which are used to create plasma discharges. Tu et al. [4] reported the treatment of NiO/Al_2_O_3_ using H_2_/Ar DBD plasma at atmospheric pressure and low temperature (<300 °C). It was found that plasma is capable of reducing NiO to Ni, increasing the surface conductivity, and modifying the discharge characteristics of the plasma. Liu et al. [5] studied the influence of non-thermal plasma on the catalytic properties of various metal-supported catalysts, such as Pt, Pd, and Ni on alumina and HZSM-5 as the supports. These authors reported that the plasma treatment could remarkably enhance the dispersion of the metal on the surface of the support, increasing the activity of the catalyst at low temperatures by generating and redistributing the acidic and basic sites, as well as improving the stability of the catalyst. It was further found that the plasma-treated catalysts show a higher resistance to carbon deposition compared to those prepared by conventional thermal synthesis methods. In addition to the modification of the surface acidity, this was attributed to an improved interaction between the metal and the support after being treated by plasma discharges, as well as to a higher dispersion of metal active sites. Karuppiah et al. [6] investigated the plasma reduction of Ni/Al_2_O_3_ and CeO_2_-Ni/Al_2_O_3_. Their results indicated that a better dispersion for metal nanoparticles can be obtained due to the treatment of the surface with DBD plasma.

The integration of plasma and catalyst aims at improving the productivity of the process towards desired products, while reducing the generation of unwanted products [7,8,9,10]. Despite the synergy of the plasma and catalyst, the formation of undesired products can still take place, which can therefore influence the overall performance of a plasma-catalyst system. As a typical instance, the plasma-driven conversion of hydrocarbons (e.g., CH_4_) usually produces carbon-containing deposits, which is considered a drawback in such processes, due to its influence on the stability of the plasma, as well as on the catalytic activity of the catalyst. Khoja et al. [11] studied dry reforming of methane in a DBD plasma reactor packed with γ-alumina. SEM images showed the formation of carbonaceous species on alumina. In addition, EDX elemental analysis detected the presence of carbon in the composition of alumina, comparably higher than the fresh γ-alumina powder. Chiremba et al. [12] performed SEM characterization of BaTiO_3_ after 80 hr exposure to CH_4_ plasma. BaTiO_3_ spheres were used as packing for the conversion of methane in a packed-bed DBD reactor. The grain structure of BaTiO_3_ was influenced after the exposure to DBD discharges, where the grains tend to agglomerate to form larger ones, resulting in the formation of a layered structure.

The conversion of CH_4_ with DBD plasma reactors is accompanied by the formation of deposits, originating from the decomposition of CH_4_ to CH_x_ fragments [13,14]. These fragments and their interaction generate other potential precursors (e.g., C_2_H, C_2_H_3_) for the formation of deposits. The integration of catalyst surfaces with the DBD plasma discharges is one of the approaches that can reduce the formation of deposits; however, some carbonaceous species are still formed on the catalyst surface. The presence of deposits can influence the stability of the operation for packed-bed DBD reactors. The characterization of the formed deposits can identify the nature of the deposits and their morphology, which gives more insight into the mechanism of the carbonaceous species formation, as well as their effect on the performance of the process.

Therefore, in this article, the deposits formed during the plasma conversion of CH_4_ for both the non-packed reactor and the reactor packed with different packing materials are characterized using high-resolution scanning electron microscopy (HR-SEM) to study the deposits’ layer, as well as the structural changes of the catalyst samples after being exposed to CH_4_+Ar DBD plasma discharges. Energy dispersive X-ray analysis (EDX) is utilized to analyse the elemental composition of the deposits to supplement the results obtained by HR-SEM.

## 2. Materials and Methods

### 2.1. Packing Materials

The following materials were utilized as the packing: γ-alumina (Alfa Aesar, 234 m^2^/gr), Pd (1 wt%)/γ-alumina (Alfa Aesar, surface area: 160 m^2^/gr), α-alumina (Alfa Aesar, 0.82 m^2^/gr), silica-SBA-15 (Sigma Aldrich, 673 m^2^/gr), MgO/Al_2_O_3_ (Sasol Company, 440 m^2^/gr), and BaTiO_3_ (Alfa Aesar, 18 m^2^/gr). The surface area of the samples, as given in the brackets above, was measured with nitrogen physisorption at 77 K with a Surface area and Porosity Analyzer (Micrometrics, Norcross, GA, USA), TriStar, Micrometrics.

### 2.2. Plasma Processing

The mixture of CH_4_ and Ar was introduced to the inlet of the DBD reactor, which was made of a quartz tube, acting as the dielectric with an i.d. of 4 mm (inner diameter) and an o.d. of 6 mm (outer diameter). A stainless steel rod with a diameter of 1.6 mm, acting as the high voltage electrode, was fixed at the centre of the quartz tube. The quartz tube was covered with a rigid stainless steel tube with a length of 10 cm, which acted as the ground electrode. In the case of the packed reactor, the powder was sieved within the particle size range of 100–300 µm and packed inside the discharge gap of the DBD plasma reactor, covering the discharge gap (1.2 mm) between the high voltage electrode and the dielectric quartz tube for the 10 cm length of the plasma discharge zone. A total flow rate of 50 mL/min of the mixture of methane and argon was applied, containing 5 vol% of CH_4_ as the reactant.

The reaction products were analysed with an online Varian 450 Gas Chromatograph (Varian Inc, Middelburg, The Netherlands) equipped with TCD and FID detectors (Varian Inc, Middelburg, The Netherlands). The products were separated by Hayesep T&Q, Molsieve 13x, and PoraBOND Q columns (Agilent, Santa Clara, CA, USA) to analyse gas-phase products. A high-voltage probe (TESTEC TT-HVP15 HF, Frankfurt, Germany), a probe for connecting the ground electrode (TESTEC TT-HV 250, Frankfurt, Germany), a 3.9 nF capacitor, and an oscilloscope (Pico Scope 2000 series, Pico Tech, Cambridgeshire, UK) were used to measure the discharge power. The discharge power was calculated from Q-V Lissajous figures. The DBD plasma was generated between the high voltage electrode and the ground electrode by applying a high voltage of 7–8 kV with a frequency of 23 kHz. The discharge power was maintained in the range of 7–8 Watt during the 2.5 h of the plasma processing. All experiments were performed at ambient conditions. The outlet temperature of the reactor was measured with a thermocouple, which was close to the ambient temperature, and therefore no heating effect was observed at the outlet of the reactor.

### 2.3. Scanning Electron Microscopy (SEM)/Energy Dispersive X-ray Analysis (EDX) Characterization

Scanning electron microscopy (SEM) with a high resolution was performed using a Zeiss MERLIN HR-SEM instrument. This instrument was also equipped with energy dispersive X-ray analysis from OXFORD Instruments for obtaining the elemental composition of the samples. The quartz tube covered with deposits on its inner surface was cut and mounted inside the chamber of the SEM instrument. For the catalyst samples, the typical SEM sample holder was used. A small amount of the catalyst powder was attached to the sample holder using carbon adhesive tape and the holders were then mounted inside the chamber. For SEM images, a low acceleration voltage of around 1 kV was applied, in order to avoid the charging effects that may occur due to the low conductivity of the deposits formed on the quartz tube. The surface charging became extreme when an acceleration voltage higher than 3 kV was applied, where it was not possible to see a clear image of the sample and, where a large white (bright) area on the image was observed instead. For energy dispersive X-rays (EDX) analysis, a higher voltage of 10 kV was applied in order to produce the emission of X-rays from the sample. It should be mentioned that CHN elemental analysis (Organic Elemental Analyzer, Flash 2000, Thermo Fisher Scientific Inc.) was performed to identify the hydrogen and carbon content of the formed deposits, constituted on the inner surface of the dielectric quartz tube.

## 3. Results and Discussion

### 3.1. SEM/EDX Characterization of Deposits Formed on the Dielectric Quartz Tube

**Figure 1:**
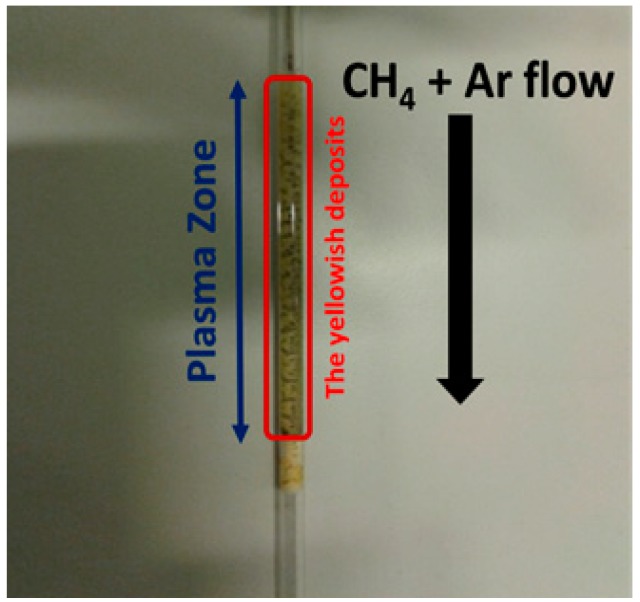
The yellowish deposits formed on the dielectric quartz tube.

A layer of yellowish deposits was formed during the plasma reaction for the non-packed reactor on the inner surface of the dielectric quartz tube, as depicted in Figure 1.

**Figure 2:**
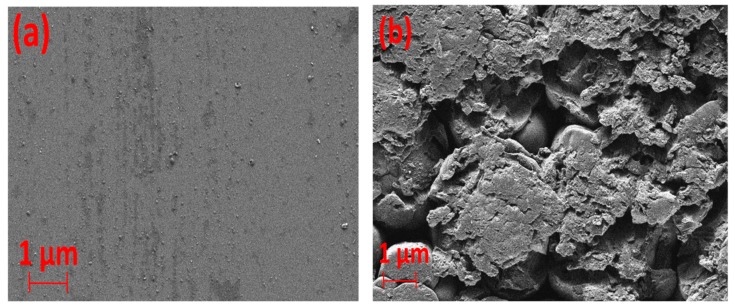
The SEM image of deposits formed on the inner surface of the dielectric quartz tube. (**a**) Before exposure to plasma (clean quartz dielectric tube) and (**b**) after exposure to plasma.

The SEM image of the layer (Figure 2b) indicates that the deposits possess an amorphous structure with irregular pores and cracks on the surface, showing a combination of both smooth areas and rough areas. For comparison, the SEM image of the clean dielectric quartz tube, before the exposure to plasma, is depicted in Figure 2a, which shows a flat surface.

**Figure 3:**
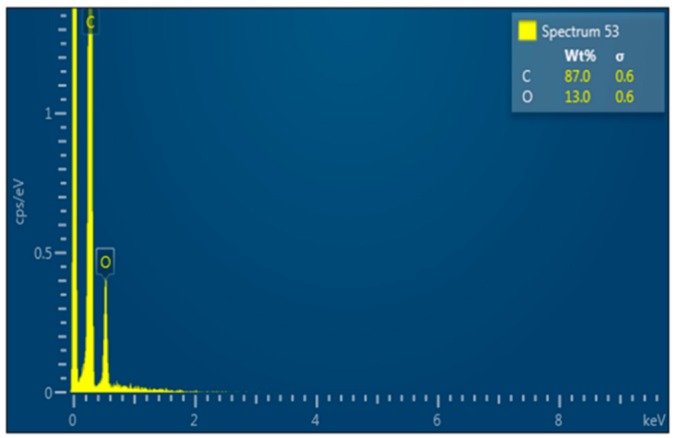
Spectra of deposits formed on the inner surface of the quartz tube.

The composition of the deposits was evaluated by EDX analysis, as depicted in Figure 3. It should be noted that it is not possible to detect hydrogen with EDX analysis and therefore, the chemical composition was analysed excluding the content of hydrogen.

The elemental analysis shows a high content of carbon in the structure of deposits, with an average of 88.6 wt% (91.2 at%) and the rest is oxygen, which either originates from the quartz tube structure (SiO_2_) or from the moisture, which usually exists in argon flow and which is difficult to avoid, even in a vacuum system. The impurity of oxygen has been reported in previous studies during the growth of plasma polymerized films using the mixture of CH_4_ and Ar in vacuum systems [15,16].

In order to include the content of hydrogen, the composition of the deposits’ layer was analysed using a CHN elemental analyser, which is capable of determining the amount of hydrogen and carbon in the composition of the deposits. It was revealed that the H/C atomic ratio for the deposits is equal to 1.7 ± 0.1. From this, it was concluded that the deposits possess a polymer-like structure, considering a higher atomic content of H (~60 at%) [17]. This could further indicate that the layer of deposits is not a highly conductive material, due to a low atomic content of carbon.

### 3.2. SEM/EDX Characterization of γ-Alumina and Pd/γ-Alumina After Exposure to DBD Plasma

The catalyst samples, which were used as packing inside the discharge gap of the implemented DBD plasma reactor, were characterized by high-resolution scanning electron microscopy. The results indicate that the structure of the catalyst samples can be influenced after being exposed to CH_4_+Ar DBD plasma. The changes on the structure of the catalyst originate from the formation of solid products on the catalyst, resulting from CH_4_ decomposition, as well as from plasma treatment of the catalyst, while being exposed to plasma-induced species (ions, electrons, radicals) upon the generation of discharges.

**Figure 4:**
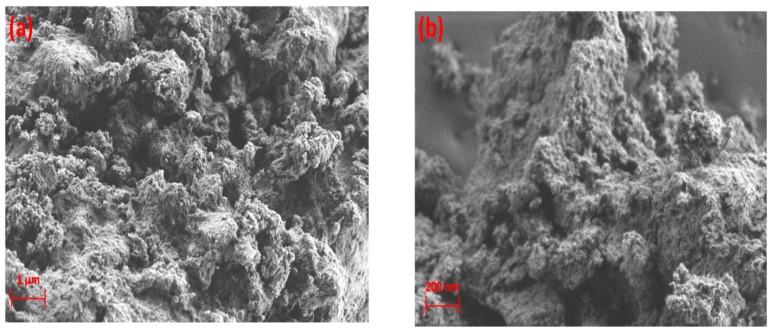

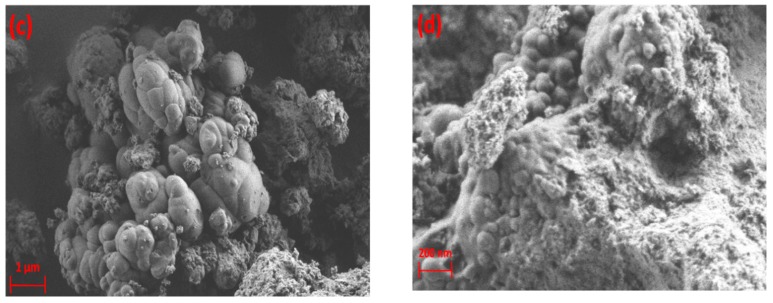
SEM images of γ-alumina (**a**,**b**) before exposure to DBD plasma; (**c**,**d**) after exposure to DBD plasma.

**Figure 5:**
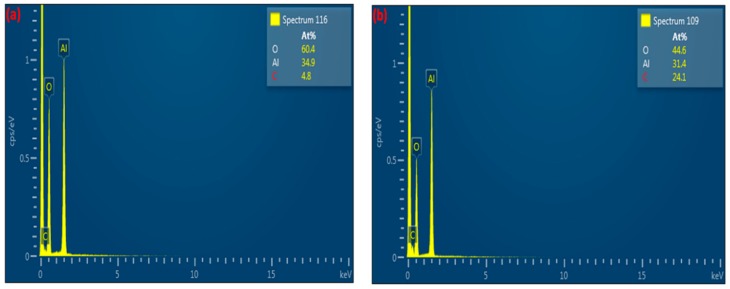
EDX spectra of γ-alumina (**a**) before exposure to DBD plasma; (**b**) after exposure to DBD plasma.

Figure 4 shows the images of γ-alumina before and after exposure to plasma. The surface of γ-alumina is covered with amorphous carbon-containing agglomerates after exposure, and has a different structure than the fresh γ-alumina sample, indicating morphological changes during the plasma reactions. The presence of carbonaceous species on the γ-alumina structure was further confirmed by EDX spectra, as depicted in Figure 5.

**Figure 6:**
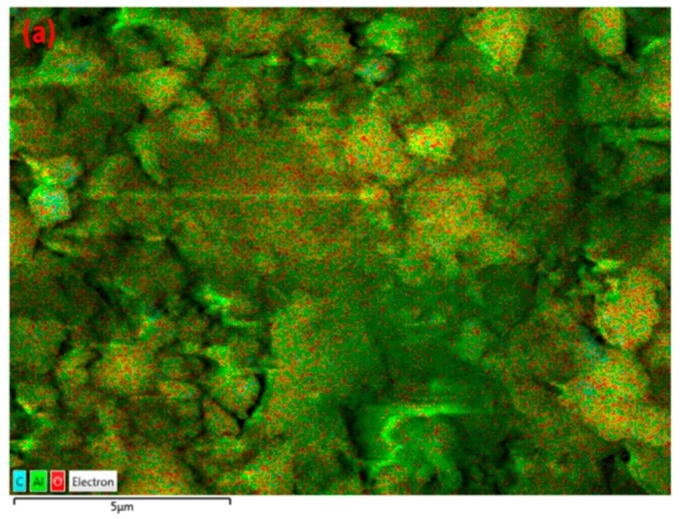

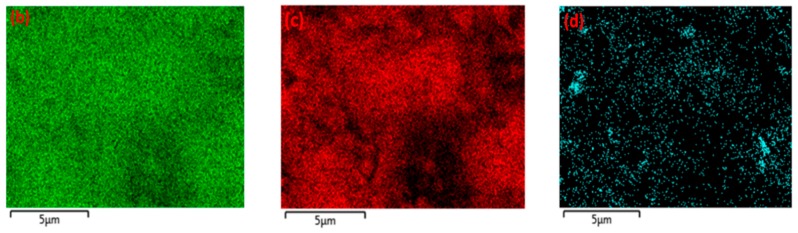
EDX mapping of γ-alumina after exposure to CH_4_+Ar DBD plasma. (**a**) Layered image; (**b**) Al map; (**c**) O map; (**d**) C map.

These results indicate that γ-alumina undergoes both structural and chemical changes during the exposure to CH_4_+Ar plasma. The carbon-containing deposits were randomly formed on the γ-alumina structure, as observed by SEM/EDX mapping analysis of γ-alumina after exposure to plasma (Figure 6d). This could further indicate that deposits did not cover the structure uniformly.

For some areas of the fresh samples (i.e., before the exposure to plasma), a small amount of carbon was detected in the EDX analysis. This may be ascribed to reagents that were used in the preparation of the catalyst, e.g., carbonate (CO_3_)^2^, which could remain as impurities in the sample. It may also originate from the carbon adhesive tape, used for sampling, which is in direct contact with catalyst particles. Regardless of the source of carbon detected in the fresh samples, the atomic percentage of carbon in the fresh samples was always significantly lower than that detected in the plasma-processed samples.

**Figure 7:**
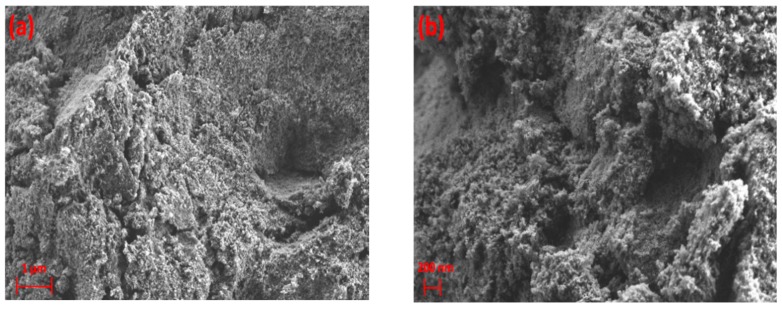

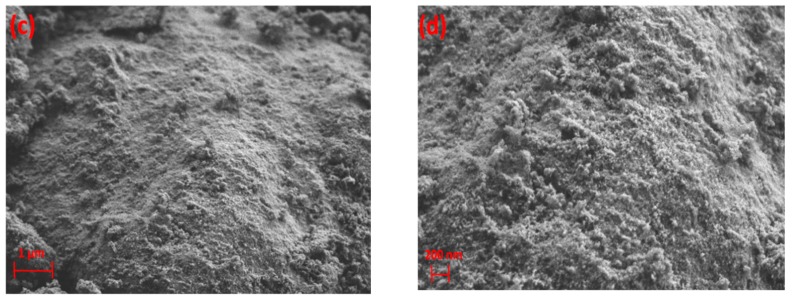
SEM images of Pd/γ-alumina (**a**,**b**) before exposure to DBD plasma; (**c**,**d**) after exposure to DBD plasma.

**Figure 8:**
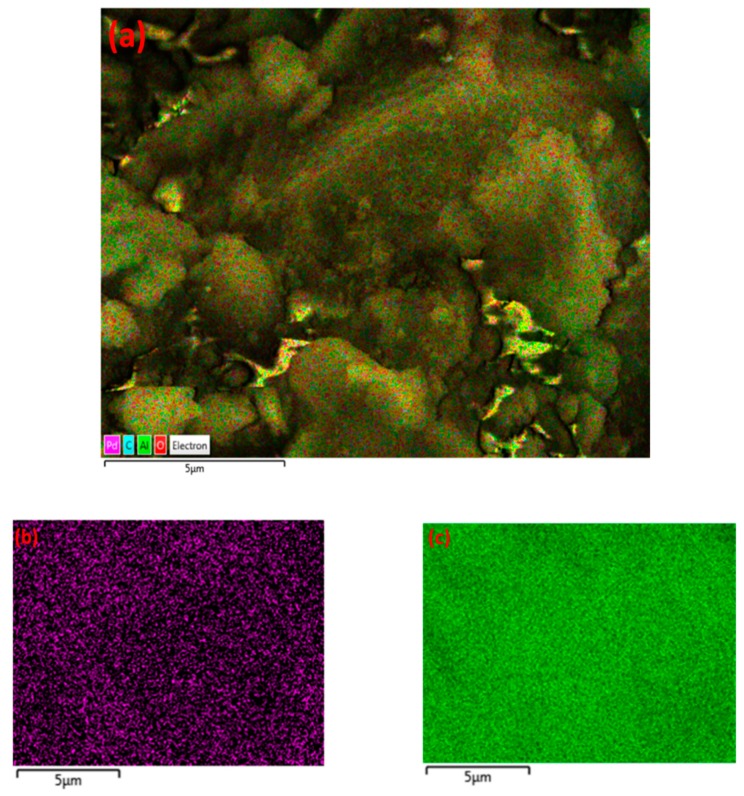

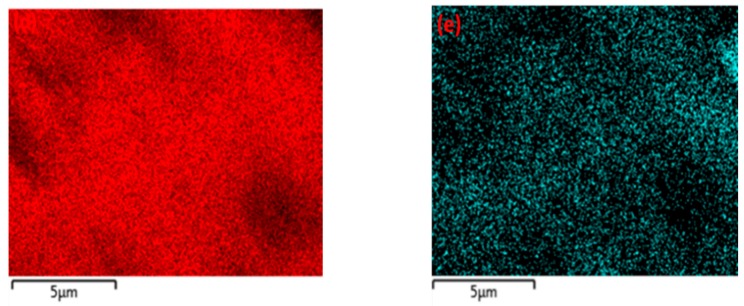
EDX mapping of Pd/γ-alumina after exposure to CH_4_+Ar DBD plasma. (**a**) Layered image; (**b**) Pd map; (**c**) Al map; (**d**) O map; (**e**) C map.

The formation of agglomerates was not observed in SEM images for Pd/γ-alumina after exposure to plasma, as shown in Figure 7. This demonstrates that the Pd-containing surface becomes resistant to the deposition of solid species, due to the presence of Pd active sites on γ-alumina in such a way that the presence of Pd particles promotes the hydrogenation of deposit precursors (e.g., CH_x_ and C_2_H_y_) on the catalyst surface, resulting in a lower amount of deposits being formed. The results of the conversion of methane and the selectivity of the deposits can be in found in Table S1 of the Supplementary Information. These results indicate that the selectivity of deposits decreases from 61.2% (i.e., for γ-alumina) to 24.2% (i.e., for Pd/γ-alumina). This therefore confirms the interaction of deposit precursors (e.g., CH_x_ and C_2_H_y_) with Pd particles, promoting their hydrogenation towards gas-phase products faster than their deposition on the catalyst surface. Despite the effect of Pd in the reduction of the deposits, the surface morphology of Pd/γ-alumina has still been influenced after exposure to plasma, where the surface has become smoother, as can be seen in Figure 7c,d, compared to a rough surface observed for the fresh sample before exposure to plasma (Figure 7a,b). The result of EDX mapping for Pd/γ-alumina (Figure 8) exhibits the presence of carbon-containing areas; however, the amount of carbon is notably lower and more scattered, which therefore could not lead to the formation of carbon-containing agglomerates.

### 3.3. SEM/EDX Characterization of High Dielectric BaTiO_3_ After Exposure to DBD Plasma

**Figure 9:**
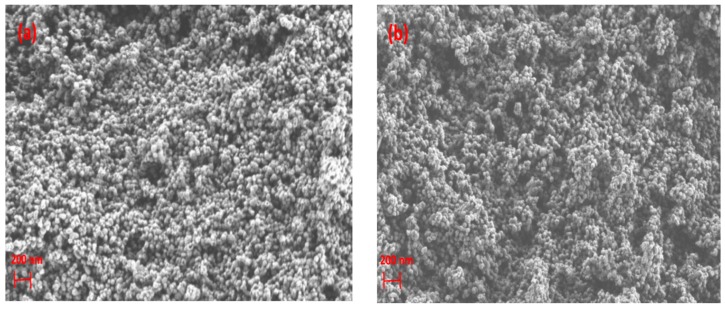
SEM images of BaTiO_3_ (**a**) before exposure to DBD plasma; (**b**) after exposure to DBD plasma.

The SEM images of BaTiO_3_ before and after exposure to plasma are shown in Figure 9a. It can be observed that the tested BaTiO_3_ has a porous structure. The high porosity of the packing increases the possibility for charges (i.e., those charges transferred between the high voltage electrode and the ground electrode) to be trapped inside the pores. In combination with the high dielectric constant of BaTiO_3_, this porous structure consequently strengthens the occurrence of partial discharging [18]. Consequently, this causes a weaker electric field at contact points of high dielectric BaTiO_3_, which leads to a lower conversion of methane.

On the other hand, a lower conversion of methane yields a lower amount of deposits. This is the main reason that the formation of agglomerates is not observed for BaTiO_3_ after exposure to CH_4_+Ar plasma, in contrast to γ-alumina. In agreement with this, the conversion of CH_4_ for the BaTiO_3_ packed reactor was 9.3%, which is remarkably lower than the 47.7% obtained for the γ-alumina packed reactor during the 2.5 h of plasma processing. In this case, a lower amount of solid products became deposited on the surface, which then lowered the probability of the formation of agglomerates during the plasma reaction. However, the exposure of BaTiO_3_ to DBD discharges could have influenced the morphology in such a way that new voids (pores) were created in the structure of BaTiO_3_. The formation of pores (voids) inside the structure of plasma-treated materials (e.g., silica) has been previously observed after the treatment of the surface by Ar plasma [19,20].

**Figure 10:**
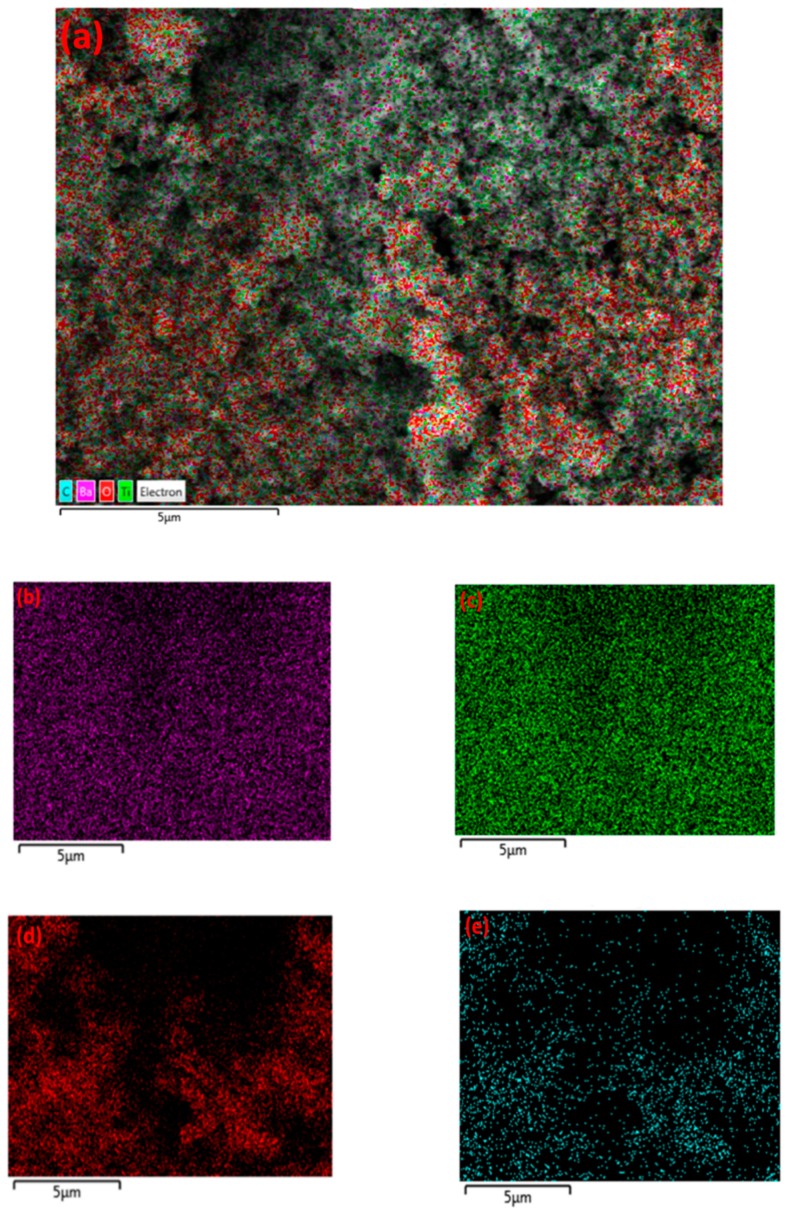
EDX mapping of BaTiO_3_ after exposure to CH_4_+Ar DBD plasma. (**a**) Layered image; (**b**) Ba map; (**c**) Ti map; (**d**) O map; (**e**) C map.

In order to see the presence of carbon in the elemental composition of BaTiO_3_, SEM/EDX mapping was performed, as shown in Figure 10. It can be seen that carbon-rich deposits do not form a dense layer on BaTiO_3_, and their presence is scattered.

### 3.4. SEM/EDX Characterization of MgO/Al_2_O_3_, Silica-SBA-15 and α-Alumina After Exposure to DBD Plasma

**Figure 11:**
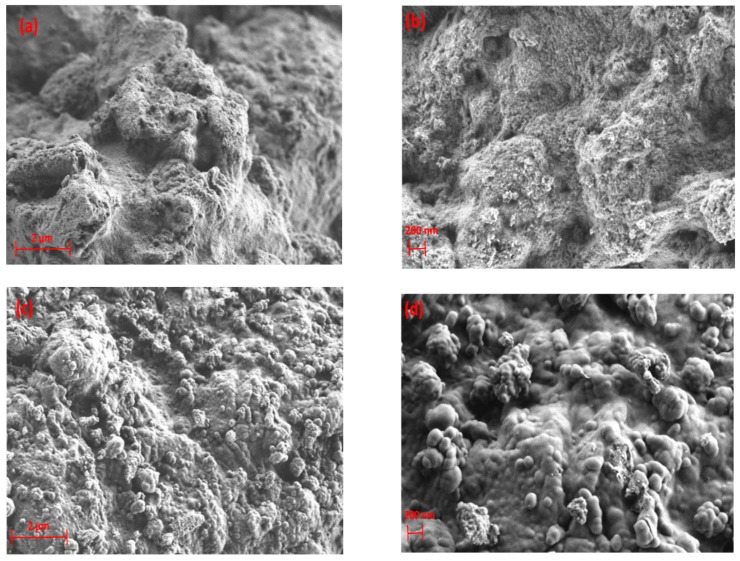
SEM images of MgO/Al_2_O_3_ (**a**,**b**) before exposure to DBD plasma; (**c**,**d**) after exposure to DBD plasma.

MgO/Al_2_O_3_, silica-SBA-15, and α-alumina were processed by exposing them to CH_4_+Ar DBD discharges. All these materials were morphologically influenced after being treated by plasma. SEM images of MgO/Al_2_O_3_ show that its structure was highly affected, where the formation of agglomerates can be clearly seen in Figure 11c,d.

**Figure 12:**
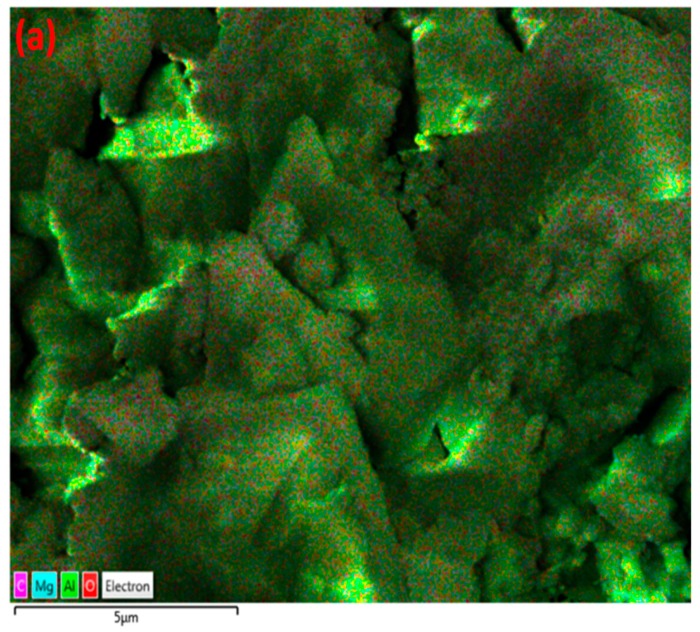

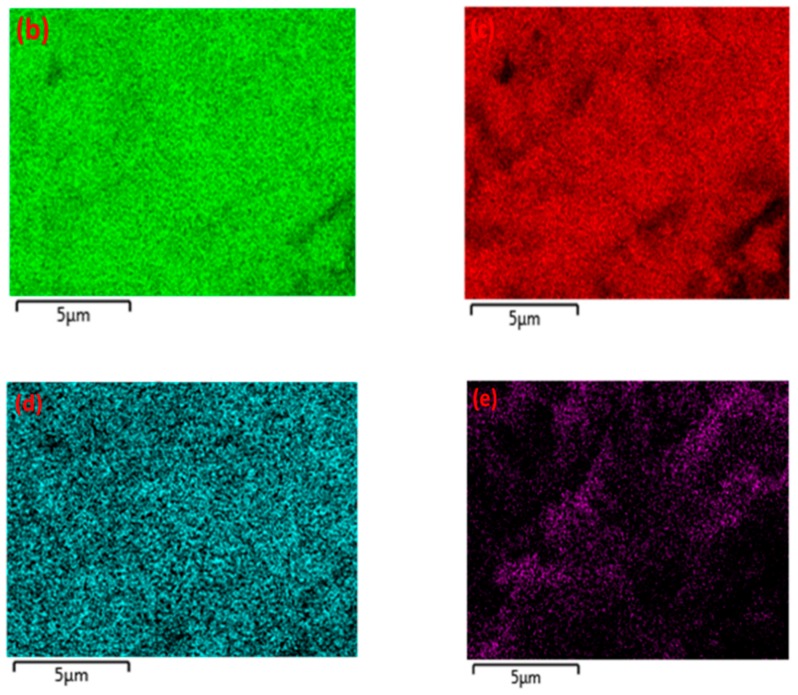
EDX mapping of MgO/Al2O3 after exposure to CH_4_+Ar DBD plasma. (**a**) Layered image; (**b**) Al map; (**c**) O map; (**d**) Mg map; (**e**) C map.

The structure of MgO/Al_2_O_3_ is influenced by the presence of carbon-rich deposits randomly, whereas the formation of agglomerates occurs regionally. This can be further seen by SEM/EDX mapping, as shown in Figure 12.

**Figure 13:**
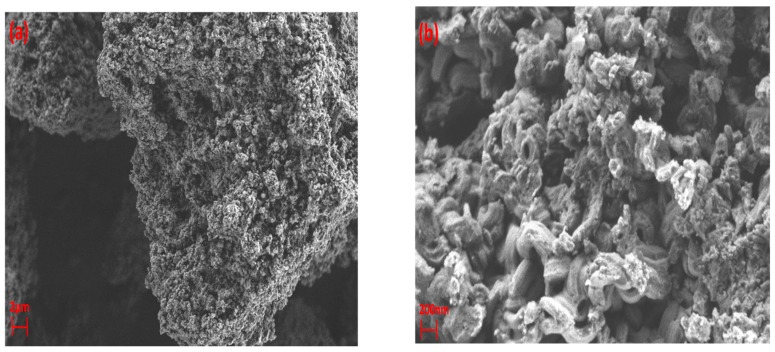

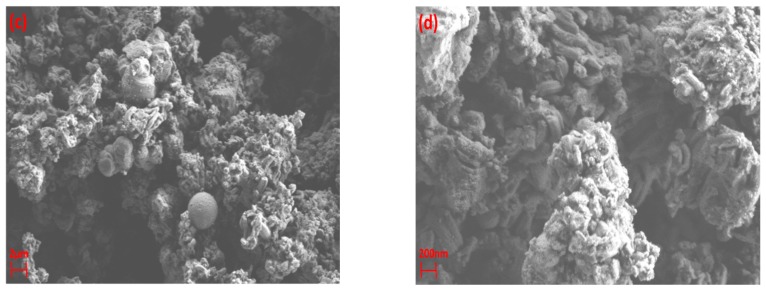
SEM images of silica-SBA-15 (**a**,**b**) before exposure to DBD plasma; (**c**,**d**) after exposure to DBD plasma.

Figure 13 depicts the morphology of silica-SBA-15 for the fresh sample and the one processed by DBD plasma. The tested silica-SBA-15 constitutes a meso-porous rope-like structure, according to Figure 13b.

**Figure 14:**
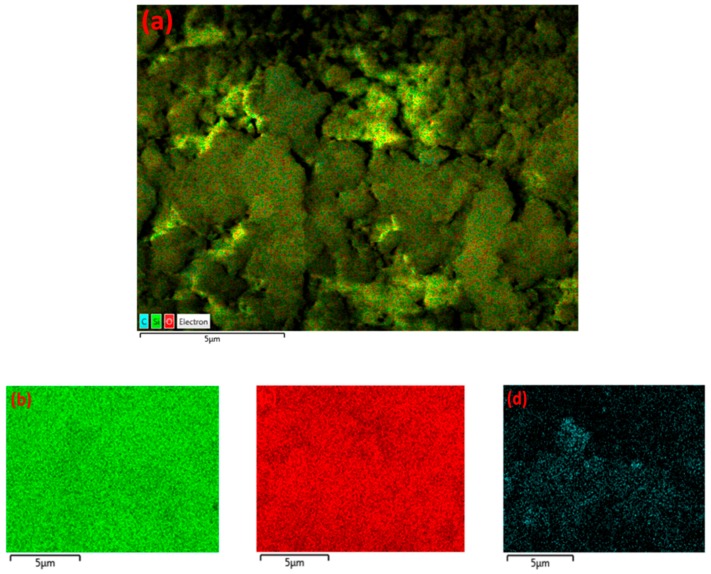
EDX mapping of silica-SBA-15 after exposure to CH_4_+Ar DBD plasma. (**a**) Layered image; (**b**) Si map; (**c**) O map; (**d**) C map.

In addition to the deposition of solid products, silica-SBA-15 undergoes structural changes, which can be seen in the form of the generation of new voids (pores) after exposure to plasma, as shown in Figure 13c. Furthermore, the scattered presence of carbon in the structure of silica-SBA-15 was observed by SEM/EDX mapping, as shown in Figure 14.

**Figure 15:**
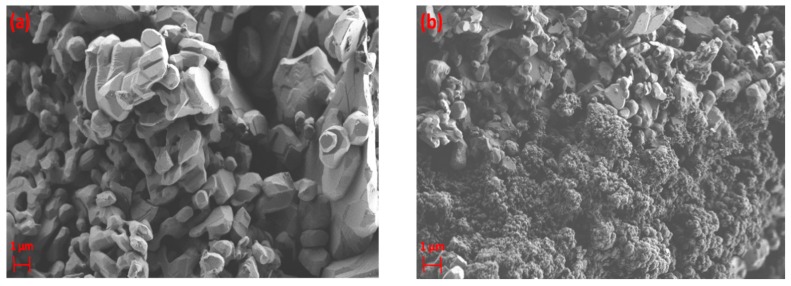
SEM images of α-alumina (**a**) before exposure to DBD plasma; (**b**) after exposure to DBD plasma.

**Figure 16:**
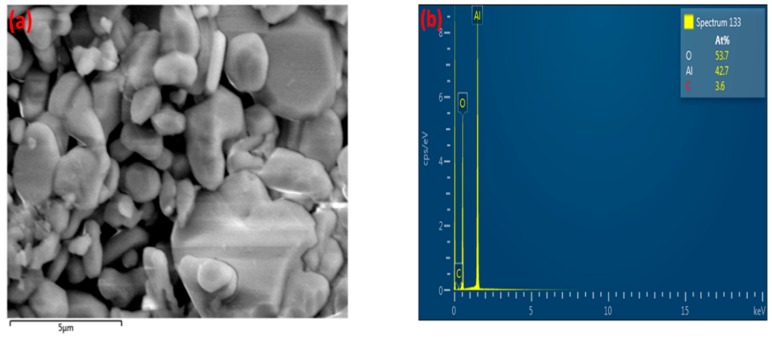

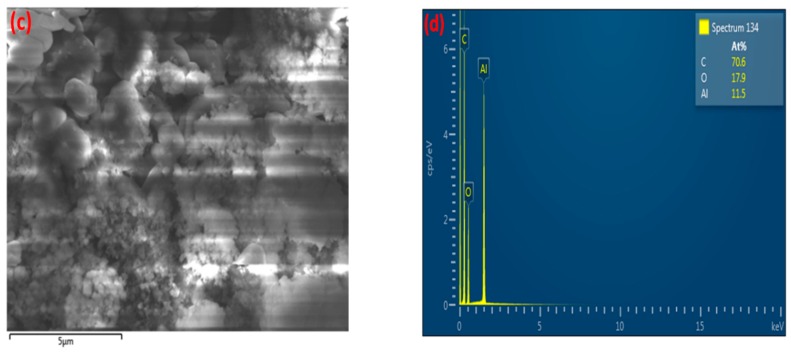
EDX electron image and elemental analysis of α-alumina sample after exposure to CH_4_+Ar DBD plasma. (**a**,**b**) Region A; (**c**,**d**) Region B.

Similarly, the formation of agglomerates was observed for α-alumina after exposure to CH_4_ plasma, as shown in Figure 15b. The fresh α-alumina possesses a crystalline structure, according to Figure 15a. This crystalline structure is partially influenced by exposure to the plasma, where the formation of agglomerates can be observed in the structure of α-alumina. Moreover, EDX results indicate that carbon-rich areas are regionally dispersed in the structure of α-alumina, as depicted in Figure 16, for two different regions of the analysed sample. The atomic percentage of carbon changes from 3.6 to 70.6 at%, respectively, for region A and region B of the analysed sample.

**Figure 17:**
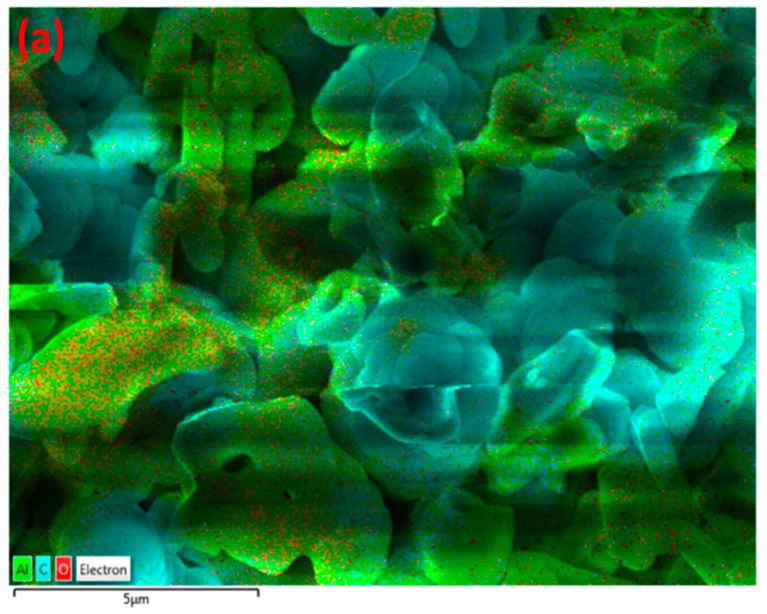

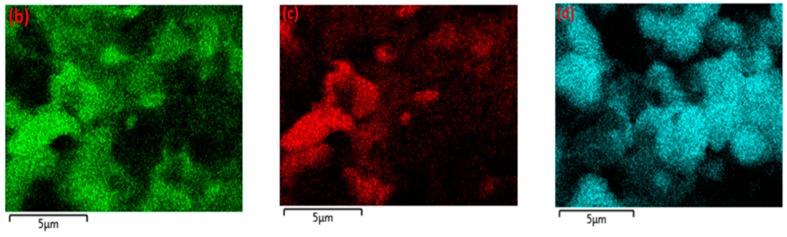
EDX mapping of α-alumina after exposure to CH_4_+Ar DBD plasma. (**a**) Layered image; (**b**) Al map; (**c**) O map; (**d**) C map.

As shown in Figure 16c, region B of the sample has been covered with carbon-containing deposits. On the contrary, region A of the sample (Figure 16a) is almost clean, similar to the structure of the fresh sample, as depicted in Figure 15a. EDX mapping of α-alumina for region B (Figure 17) also shows the coverage of α-alumina with carbonaceous species in the form of agglomerates.

These results indicate that all catalyst supports undergo morphological changes. This includes materials such as MgO/Al_2_O_3_ (440 m^2^/gr) and silica-SBA-15 (673 m^2^/gr), which both possess high surface areas, as well as γ-alumina, which possesses a relatively lower surface area (234 m^2^/gr). Even the structure of a low surface area material (i.e., α-alumina) is influenced by the formation of deposits. The results of the conversion of CH_4_ and the selectivity of the deposits (Table S1) show a similar range of deposits’ selectivity for catalyst supports with various surface areas. Therefore, according to these results, no specific correlation between the surface area and the deposits’ formation can be drawn for the plasma catalytic conversion of CH_4_. Further investigations may be conducted to study the influence of plasma treatment, as well as deposits on the surface area and porosity of the packing. This is beyond the scope of the present article and therefore can be a topic of future research.

## 4. Conclusions

High-resolution scanning electron microscopy (HR-SEM) was utilized to characterize the deposits formed during the CH_4_+Ar DBD plasma. A low voltage of 1 kV was applied in order to prevent the phenomenon of surface charging, which was intensively observed for high voltages (≥3 kV). This could furthermore confirm the low conductivity of the deposits. For the deposits formed on the dielectric quartz tube, the SEM image exhibited an amorphous structure, where the morphology remained unchanged throughout the length of the plasma zone. EDX analysis indicated that the chemical composition of the deposits mainly consists of carbon (~91 at%), although it was noted that this chemical composition is estimated excluding the presence of hydrogen, as the EDX detector is not able to analyse hydrogen. The presence of hydrogen was identified with CHN elemental analysis, indicating that the H/C molar ratio of the deposits layer was 1.7 ± 0.1. This therefore indicated the polymeric nature of the formed deposits, due to a high content of H (~60 at%). The structure of γ-alumina was influenced after exposure to plasma by the formation of regionally amorphous carbon-containing agglomerates. On the contrary, the structure of Pd/γ-alumina did not exhibit the presence of carbon-rich agglomerates, indicating the resistance of the surface to deposits’ formation due to the presence of Pd particles, which could promote the hydrogenation of deposit-precursors faster than their deposition to solid species. All other plasma-processed materials, including BaTiO_3_, MgO/Al_2_O_3_, silica-SBA-15, and α-alumina, clearly showed morphological changes after being exposed to CH_4_+Ar plasma, not only due to the formation of deposits, but also owing to the plasma treatment of the surface, which could influence the structure of these materials.

## Supplementary Materials

The following are available online at https://www.mdpi.com/2079-4991/9/4/589/s1, Table S1. The conversion of CH_4_ and the selectivity of gas-phase (C_2+_) products and deposits for the non-packed (Blank) and the packed DBD plasma reactor.
