# Supplementary material for: High-Resolution SEM and EDX Characterization of Deposits Formed by CH4+Ar DBD Plasma Processing in a Packed Bed Reactor
Source: Nanomaterials (Basel). 2019 Apr 10;9(4):589. doi: 10.3390/nano9040589 (PMC6523143; doi:10.3390/nano9040589)
Supplement: Supplementary file 1 [file nanomaterials-09-00589-s001.pdf]

High resolution SEM and EDX characterization of deposits formed by CH_4+Ar DBD plasma processing in a packed bed reactor

Mohammadreza Taheraslani[1,2*] and Han Gardeniers[1]

1.Mesoscale Chemical Systems, MESA+ Institute for Nanotechnology, University of Twente, P.O. Box 217, 7500 AE Enschede, The Netherlands.
2.Faculty of Science and Technology, University of Twente, P.O. Box 217, 7500 AE Enschede, The Netherlands.
[*]Correspondence: m.taheraslani@utwente.nl

Supplementary Information

Table S1. The conversion of CH_4 and the selectivity of gas-phase (C_{2+}) products and deposits for the non-packed (Blank) and the packed DBD plasma reactor. Total flow rate=50 ml/min, CH_4 concentration=5 %vol CH_4, Voltage = 7-8 kV, f = 23 kHz, P = 7-8 W.

Experiment	CH_4 Conversion %	C_{2+} Selectivity %	Deposits Selectivity %
Blank	39.2	49.7	50.3
γ-alumina	47.7	38.8	61.2
Pd/γ-alumina	32.8	75.8	24.2
$BaTiO_3$	9.3	76.4	23.6
MgO/Al_2O_3	35.5	42.1	57.9
Silica-SBA-15	33.4	49.4	50.6
α-alumina	46.7	37.8	62.2

$$\text{Conversion of } CH_4\ (\%) = \frac{CH_4 \text{ converted (mmol/s)}}{CH_4 \text{ introduced (mmol/s)}} \times 100$$

$$\text{Selectivity of } C_{2+} \text{ gas-phase products } (\%) = \frac{\sum C_xH_y \text{ produced (mmol/s)} \times x}{CH_4 \text{ converted (mmol/s)}} \times 100$$

$$\text{Selectivity of the formed deposits } (\%) = \frac{CH_4 \text{ converted (mmol/s)} - CH_4 \text{ consumed to form gas-phase products (mmol/s)}}{CH_4 \text{ converted (mmol/s)}} \times 100$$
